# Chronic Stress Activates PlexinA1/VEGFR2-JAK2-STAT3 in Vascular Endothelial Cells to Promote Angiogenesis

**DOI:** 10.3389/fonc.2021.709057

**Published:** 2021-08-16

**Authors:** YanJie Lu, HanZheng Zhao, Ying Liu, YanZhen Zuo, Qian Xu, Lei Liu, XiaoMin Li, HongBin Zhu, Ying Zhang, Shuling Zhang, XiangYang Zhao, YuHong Li

**Affiliations:** ^1^ Department of Pathology, Chengde Medical College, Chengde, China; ^2^Cancer Research Laboratory, Chengde Medical College, Chengde, China; ^3^Department of General Surgery, The Second Affiliated Hospital of Harbin Medical University, Harbin, China; ^4^Department of Psychology, Chengde Medical College, Chengde, China; ^5^Department of General Surgery, The 983rd Hospital of the Joint Service Support Force of Chinese People’s Liberation Army, Tianjin, China; ^6^Department of Laboratory, Chengde County Hospital, Chengde, China

**Keywords:** chronic stress, VEGF, PlexinA1, VEGFR2, vascular endothelial cell, angiogenesis

## Abstract

It is known that chronic stress modulates multiple processes in a complex microenvironment, such as angiogenesis and immune function. However, the role of chronic stress inducing tumor angiogenesis and how it contributes to tumor progression are not quite clear. The following study assess psychological state from numerous ambulatory cancer cases (n=332), and chronic stress-related hormone levels were further measured. Here, we show that chronic stress not only causes behavioral changes in human, most importantly attributed to an elevated level of stress-related hormones. To address this, isoprenaline, the agonist of β2-adrenergic receptor (β2-AR), was utilized for simulating chronic stress and demonstrating the mechanism of stress in tumor angiogenesis at molecular level both *in vivo* and *in vitro*. As suggested by this study, isoprenaline promote VEGF autocrine of HUVECs, which can induce plexinA1 and VEGFR2 expression. Moreover, we show that isoprenaline promoted the expression of p-JAK2 and p-STAT3 *in vitro*. The results reveal that, isoprenaline enhances the autocrine of VEGF in HUVECs and up-regulating plexinA1 and VEGFR2 levels, thus activating the phosphorylation of JAK2-STAT3 pathway, the two essential parts during angiogenesis. The present work indicates that, the mechanism of chronic stress in enhancing angiogenesis is probably achieved through activating the plexinA1/VEGFR2-JAK2-STAT3 signal transduction pathway within HUVECs, and this is probably a candidate target for developing a strategy against angiogenesis in cancer.

## Introduction

After being diagnosed as a malignant tumor, most clinical tumor patients will have a variety of psychological stress such as shock, denial, anxiety and fear. Also, surgery and chemoradiotherapy process caused by pain, nausea, vomiting, infection and other side effects will also promote the patient disappointment, anger and depression psychological problems (1, 2). Recent evidence suggests that chronic stress acts as risk factors to promote progression of multiple tumour types by facilitating tumour cells growth, increasing expression of migration or invasion-related genes (3) and promoting angiogenesis (4). As indicated in some works, catecholamine’s can activate the β2-adrenergic receptors (β2-AR), thus mediating tumour cells and tumour microenvironment (5, 6). Nonetheless, little is known about the exact mechanism of angiogenesis induced by stress in the context of tumour. This work mainly aimed to explore the precise mechanism by which isoprenaline, the β2-AR agonist, participated in the tumour angiogenesis.

Tumour angiogenesis shows tight correlation with dismal prognosis. Typically, angiogenesis indicates that new blood vessels are formed, and this exerts an important part in tumour development or metastasis (7). These new blood vessels not only provide nutritional basis but also provide conditions for distant spread of tumour cells. Tumour angiogenesis involves multiple steps, including the associations of tumour cells with vascular endothelial cells (VECs) *via* certain growth factors together with corresponding receptors, and pro-angiogenic signal transduction pathway activation. As a matter of fact, treatments against angiogenesis that target VEGFs along with specific receptors have emerged recently (8). Unfortunately, the efficacy of such drugs is not ideal, especially for advanced cancer cases; and further research is warranted for determining the anti-angiogenic drug effect (9).

A past III study has targeted VEGF receptor2 (VEGFR2) in a second-line treatment among advanced gastric cancer cases (10, 11). Nonetheless, this treatment cannot be completely translated to a superior survival to that attained by standard treatment, as suggested by randomized clinical trials (12). Thus, who is to blame the failure of VEGFR2-targeted therapy must be explored.

PlexinA1, as a transmembrane protein, can not only bind to its ligand Sema3A, Sema3C, Sema6D but also play a biological role independently (13). Recently, plexinA1 has been identified to exert a vital part in tumour biology, like angiogenesis or cell survival (14). It is interesting that, plexinA1 and VEGFR2 can produce a complex after being triggered by Sema6D in the process of heart morphogenesis (15). Our previous study detected the microvessel density within gastric cancer and reported the positive correlation between plexinA1 and angiogenesis (16). plexinA1 and VEGFR2 are co-localized within VECs of gastric cancer (17). therefore, we also examined the relationship between plexinA1 and VEGFR2 in tumour angiogenesis mediated by chronic stress. This work emphasized VEGFR2 and plexinA1 levels as well as their functional association in tumour angiogenesis induced by stress.

## Materials and Methods

### Cancer Patients and Blood Samples

335 patients were provided by the First Affiliated Hospital of Chengde Medical University who suffer from malignancy. There are 332 (99.1%) valid questionnaire and 3 (0.9%) invalid questionnaire. Inclusion criteria included: (1) Patient were pathologically diagnosed with cancer; (2) 18-80 years of age; (3) Patient had not taken antidepressants; (4) Patient had not any condition that would impede understanding of the study or the informed consent. Exclusion criteria included: (1) Patients with severe mental illness and cognitive dysfunction; (2) Patients who refuse to participate in the research and cannot communicate with the researchers normally; (3) The expected life is less than 3 months; (4) Patients with hearing and language impairment. The results of Self-Rating Anxiety and Self-Rating Depression in 215 patients with different types of cancer are available in **Table S1**. 215 blood samples were collected with the consent of the patients from the above 332 patients.

### Zung Self-Rating Anxiety Scale and Zung Self-Rating Depression Scale

The Zung Self-Rating Anxiety Scale (ZSAS) was adopted for assessing the anxiety severity, whereas the Zung Self-Rating Depression Scale (ZSDS) was employed for evaluating depression severity. The SAS and SDS index scores are then categorized into 2 levels respectively: no significant psychopathology (SAS Index <50 and SDS Index <53); presence of varying degrees of anxiety (SAS Index ≥50) or depression (SDS Index ≥53). The Zung score does not necessarily give the clinical diagnosis of anxiety or depression, instead, it suggests the severity of the clinically significant symptoms.

### Cell Culture and Transfection

Human umbilical vein endothelial cells (HUVECs), as well as human gastric cancer cell lines MGC803, were provided by Chinese Academy of Military Medical Sciences. All these cells were cultivated within the RPMI-1640 medium that contained 10% FBS. hCMEC/D3 was purchased from SCIENCE CELL Research Laboratories. hCMEC/D3 cells were cultivated within the Endothelial Cell Medium (ECM, Cat.No.1001). HUVECs and hCMEC/D3 were treated with β2-AR agonist, Isoprenaline (ISO) and β2-AR blocker, ICI 118,551 hydrochloride (ICI, Sigma-Aldrich, St. Louis, MO, USA).

Liposomes shRNA vectors carrying the shRNA sequences were purchased from Gene Pharma (Shanghai, China), the sequence is in **Table 1**. HUVECs were transfected with shplexinA1 and shNC (Negative control) with Polyplus Transfection (Strasbourg, France).

**Table 1:** Primer sequences and interference of sequence used in this study.

Gene	Sequences
shplexinA1	CCGGGCACTTCTTCACGTCCAAGATCTCGAGATCTTGGACGTGAAGAAGTGCTTTTTG
plexin-A1	5’-TGGACGACCTGTTTGAGACCA-3’ (Sense) 5’-TGATCACGTTCACCCAGAAGC-3’ (Antisense)
VEGFR2	5’-CTACCAGTACGGCACCACTCAA-3’ (Sense) 5’-TCTTCCTCCAACTGCCAATACC-3’ (Antisense)
GAPDH	5’-GAAGGTCGGAGTCAACGGAT-3’(Sense) 5’-CTGGAAGATGGTGATGGGATT-3’ (Antisense)

### Cell Proliferation Assay

HUVECs were seeded at a density of 1 × 10^4^ cells/well in a 96-well flat-bottom plate and treated with different concentrations of ISO and ICI. HUVEC proliferation was measured using CCK8 cell proliferation assay kit (Beijing Solarbio Science & Technology Co., Ltd., china) according to the manufacturer’s specifications after 0, 6, 12, 24 and 48 h of incubation, and the absorbance was measured at 450 nm using a microplate reader.

### qRT-PCR

The RNeasy Mini Kit (Invitrogen) was utilized for extracting total RNA. Then, 2 µg total RNA extracted was collected to prepare cDNA by the use of M-MLV reverse transcription system kit (Promega, Madison, WI, USA). All PCR primers sequences are available in **Table 1**. The SYBR Green qPCR Detection System (Promega, Madison, WI, USA) was utilized for qRT-PCR following specific protocols. The comparative threshold cycle approach was employed for result analysis, and GAPDH was used to be the internal reference. In the meantime, the 2^-ΔΔCt^ formula was adopted to determine fold change (FC) in mRNA expression.

### Western Blot Analysis

The cold RIPA buffer (Thermo Scientific, Rockford, IL, USA) was utilized for cell lysis. Minute (TM) Cytoplasmic and Nuclear Fractionation kit (SC003) was used to extract nucleoprotein. Thereafter, we used the BCA Protein Assay kit (Beijing Solarbio Science & Technology Co., Ltd., china) for quantifying protein content. After separating proteins through SDS-PAGE, they were transferred on the PVDF membranes. Then, each membrane was subjected to overnight incubation using primary monoclonal antibodies, including mouse anti-VEGFR2, rabbit anti-plexinA1 and mouse anti-gapdh (all Abcam, Cambridge, MA, UK), rabbit anti-Histon H3, rabbit anti-JAK, rabbit anti-STAT3, rabbit anti-p-JAK and rabbit anti-p-STAT3 (all ABclonal Co., Ltd,Wuhan, China) as well as mouse anti-β-actin (Cell Signaling Technology, Danvers, MA, USA) antibodies under 4°C. Afterwards, HRP-labeled secondary antibodies (Santa Cruz, CA, USA) were further used to incubate the membranes for 2 h. In addition, the enhanced chemiluminescence (ECL) substrate (Pierce, Rockford, IL, USA) was used for detecting protein blots, whereas the Image analysis software was employed for visualization.

### ELISA Assay

Blood samples from cancer patients were collected for 15 min of centrifugation at 3000 ×g under 4°C. Human serum catecholamine and adrenaline, as well as VEGF extracellular of HUVEC levels were examined using the corresponding human ELISA kit (Mlbio biotech company, Shanghai, China) in accordance with specific protocols. In brief, the microplate reader was used to measure absorbance 450 nm.

### Endothelial Cell Transwell Assay

In this experiment, the CORING Transwell chambers NO:3422 (Tewksbury, MA, UK) were used to quantify HUVEC migration. 200μl HUVEC (2×10^5^cells/ml) was seeded with serum free medium in the upper chambers, and chemoattractant in the bottom layers was RPMI-1640 that contained 10% FBS. After being cultured under 37°C for 24 h, we utilized cotton swabs for removing cells on surface of the upper chamber, whereas those penetrating the membrane were subjected to fixation and crystal violet staining. We selected 5 fields of view (FOV) to calculate the membrane-penetrating cell number.

### Endothelial Cell Tube Formation Assay

The Matrigel Basement Membrane Matrix with high concentration was provided by BD Biosciences (Bedford, MA, UK). Add 50μl extracellular matrix gel to each well of pre-chilled 96-well plates, incubated in 37°C for at least 30min. HUVECs suspension (2×10^4^ cells/ml) were seeded onto the solidified gel for 12h. The inverted microscope was used to examine the endothelial tube structures, while Image-Pro Plus software was employed to quantify tubing area.

### *In Vivo* Tumorigenicity Assay

The BALB/c nude mice (male, aged 6 weeks, 16–20 g) were provided by Charles River Laboratories (Beijing, China) and raised within the individually ventilated caging systems. 200 μl MGC803 gastric cancer cells (1×10^6^cells/ml) were resuspended, then the mice were given subcutaneous injection of suspension in posterior region. Mice were given intraperitoneal injection with 5mg/kg/day ISO or 0.2 mg/kg/day ICI. The same amount of normal saline was injected to controls. Mice were sacrificed after 4 weeks.

### Laser Doppler Blood Perfusion of Subcutaneous Tumour Formation in Mice

Mice of subcutaneous tumorigenesis were anesthetized with 2% isoflurane dissolved within 100% oxygen at 1 L/min until they are unresponsive to external stimuli. Then mice were put onto the heated surface at 37°C with a continuous flow of isoflurane to ensure blood perfusion will not markedly be affected by temperature changes. Each mouse was placed onto the non-reflective light-absorbing plat in the prone position. Then, the laser Doppler imager together with acquisition software was initiated.

### Statistical Analysis

All assays were carried out in triplicates. Results were expressed in the manner of means ± SD. Student’s t-test and One-way ANOVA were utilized for statistically analyzing data. A difference of P<0.05 indicated statistical significance, as marked with asterisks in all figures.

## Results

### Prevalence of Anxious and Depressive Symptoms According to the ZSAS and ZSDS

**Figure 1:**
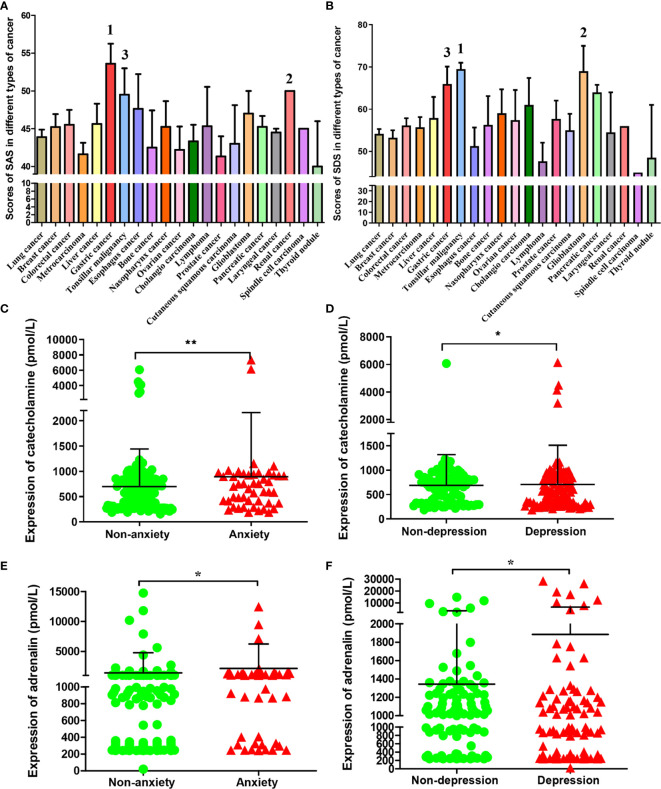
SAS, SDS and chronic stress-related hormones levels in patients with different types of cancer. The average score of SAS **(A)** and SDS **(B)** in patients with different types of cancer. The cancer types of the top three in patients with SAS and SDS scores were marked respectively. The catecholamine **(C)** and adrenaline **(E)** concentrations in blood of cases who had or had no anxiety; The catecholamine **(D)** and adrenaline **(F)** concentrations in blood of cases who had or had no depression. Data represent mean ± SD (No anxiety group, n = 171, Anxiety group, n = 48; No depression group, n = 93; Depression group, n = 113; *P < 0.05, **P < 0.01).

A total of 332 (99.1%) patients completed the questionnaire. Among them, 24.7% of the cancer patients scored to be described as anxious, 57.5% were depressed according to those criteria. When combined into an anxiety-depression composite, the prevalence was 18.7%. There were 205 (61.7%) patients with negative emotions of anxiety or depression among the total sample size. The clinicopathological diagnosis and self-evaluation scores of the 215 tumor patients were shown as **Table S1**. The SAS and SDS index of these patients were statistically analyzed according to different types of tumor. We found that the top three tumors with average SAS index were gastric cancer, renal cancer and tonsillar malignancy, (**Figure 1A**) and the top three tumors with average SDS index were tonsillar malignancy, glioblastoma and gastric cancer. (**Figure 1B**) The results showed that the anxiety and depression index of gastric cancer patients was the top three in all the cancers we detected.

### Anxiety and Depression in Cancer Patients Promote the Levels of Catecholamines and Adrenaline in Blood

According to index scores of ZSAS and ZSDS, these cancer patients were categorized into non-anxiety, anxiety and non-depression, depression group. The levels of chronic stress-related hormones in peripheral blood were measured. As a result, catecholamine contents markedly elevated among patients with anxiety and depression. (**Figures 1C, D**) Similarly, adrenaline had been found to increase significantly in patients with anxiety and depression. (**Figures 1E, F**)

### Isoprenaline Promotes Intratumoral Blood Perfusion Values

**Figure 2:**
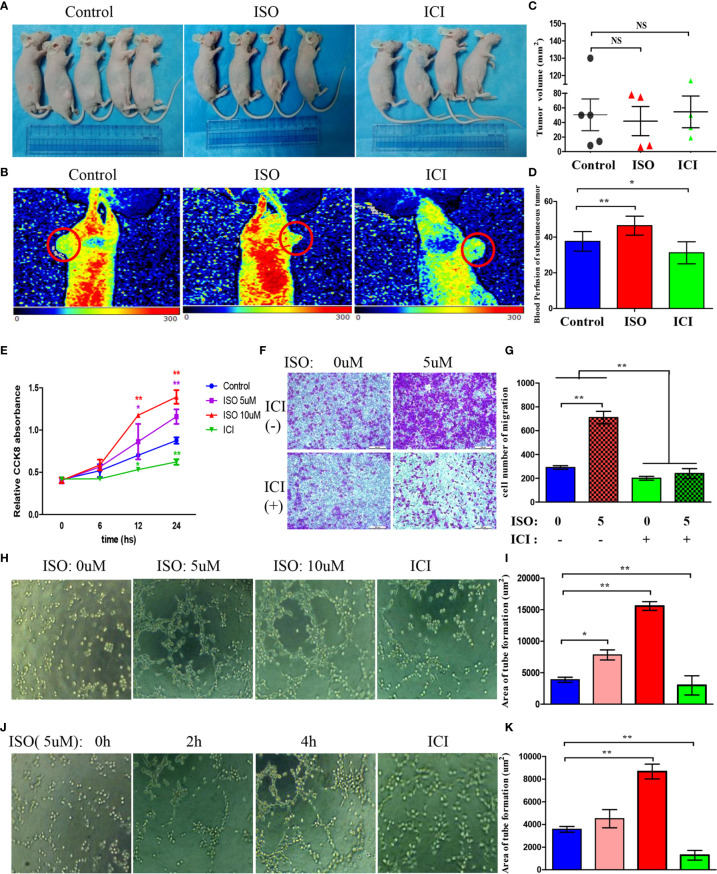
Isoprenaline promotes tumour angiogenesis *in vivo* and *in vitro*. **(A)** MGC803 cells used for subcutaneous tumour inoculation into BALB/c nude mice. ISO and ICI treatments of mice. **(B)** Representative pictures of blood perfusion in BALB/c nude mice are shown, and the blood perfusion at the tumor growth site is circled in red. **(C)** The volume of xenografts is shown. **(D)** The data of the recorded ROI perfusion values. (Control group, n = 5; ISO group, n = 4; ICI group, n = 4) **(E)** Effects of ISO and ICI on HUVECs proliferative ability. **(F)** Representative photographs of migration are shown in each panel. **(G)** Cells were counted in random five fields, and quantitative analysis is presented in every panel. **(H)** HUVECs were treated with 0, 5 or 10μM ISO and 10μM ICI within Matrigel-coated 96-well plate. **(J)** HUVECs were treated with 5μM ISO for 0, 2, 4h and 10μM ICI within Matrigel-coated 96-well plate. **(I, K)** Quantitative analysis of tube formation in random five fields is shown. Data represent mean± SD (NS, No Significance; *P < 0.05, **P < 0.01).

To examine whether chronic stress affects *in vivo* tumour formation, MGC803 cells were inoculated to mice subcutaneously. (**Figure 2A**) However, xenograft volume is not significantly different in ISO and ICI group. (**Figure 2C**) This finding consistent with a previous study by Caroline P. Le1, which suggested no influence of chronic stress on the growth of primary tumour (3). For assessing how stress affected the angiogenesis in tumour, we used Laser Doppler Blood Perfusion to quantify the levels of blood perfusion (**Figure 2B**). Then data of the recorded ROI perfusion values were evaluated by the software, and the mean of blood perfusion values was showed in **Figure 2D**. Collectively, these findings indicated isoprenaline acting on angiogenesis by effecting intratumoral blood perfusion values.

### Isoprenaline Promotes Proliferation, Migration and Tube Formation of HUVECs

Angiogenesis requires vascular endothelial cells proliferation, migration and tube formation. Therefore, we treated HUVECs with 5, 10 uM ISO or 10 uM ICI for 6, 12, 24 hours. Isoprenaline markedly increased the ability of proliferation in HUVECs. In contrast, incubated with ICI reduced the ability of proliferation. (**Figure 2E**) Transwell assay showed that treatment of 5 uM isoprenaline promoted the migration of HUVECs, which was suppressed by ICI. (**Figures 2F, G**) Isoprenaline at growing concentrations and time was used to incubate HUVECs, in addition, there is another ICI incubated group. As suggested by these findings, isoprenaline promoted HUVECs tube formation depending on its concentration (**Figure 2H**) and treatment time (**Figure 2J**), whereas the increase was reversed by ICI incubated. The area statistics of tube formation of HUVECs are shown in **Figures 2I–K**. Collectively, these findings indicated isoprenaline acting on angiogenesis by effecting vascular endothelial cells proliferation, migration and the tube formation of HUVECs, which can be reversed by ICI.

### Isoprenaline Promotes VEGF Autocrine of HUVECs, Which Can Induce plexinA1 and VEGFR2 Expression

**Figure 3:**
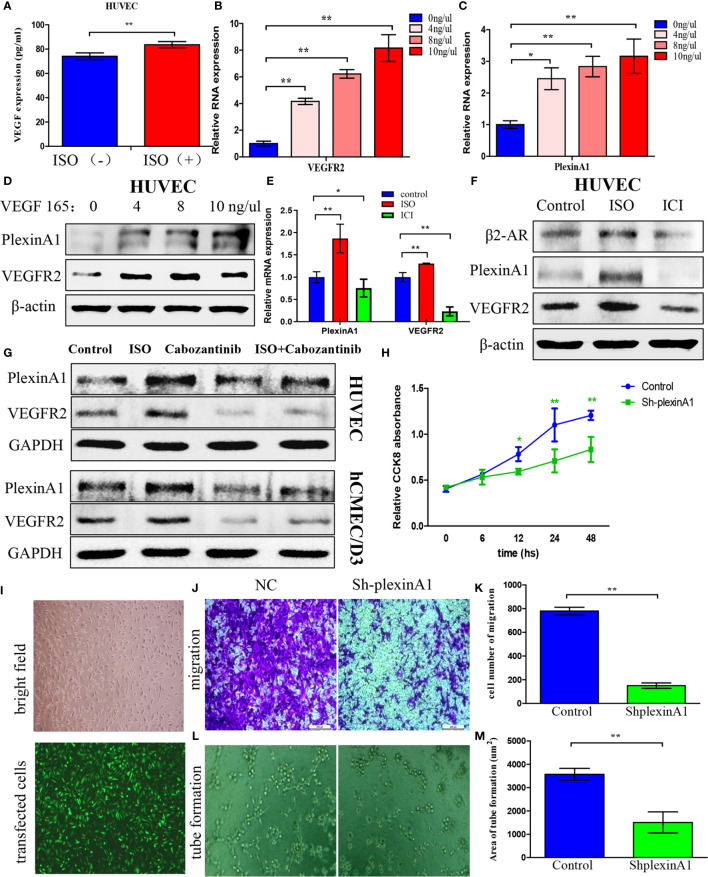
Isoprenaline promotes VEGF autocrine of HUVECs, which can induce plexinA1 and VEGFR2 expression. **(A)** HUVECs were exposed to 5μM ISO for 4 h Elisa assay revealed VEGF autocrine of HUVECs. **(B–D)** HUVECs were treated with 0, 4, 8, 10 ng/ml of VEGF165 for 24h. The relative VEGFR2 **(B)** and plexinA1 **(C)** mRNA levels were detected by qRT-PCR, and their protein levels were detected through WB **(D)**. **(E, F)** HUVECs were treated with ISO or ICI. The relative mRNA **(E)** and protein **(F)** levels of β2-AR, VEGFR2 and plexinA1 were analyzed. **(G)** HUVECs and hCMEC/D3 were treated with ISO or Cabozantinib. The protein levels of VEGFR2 and plexinA1 were analyzed by WB. **(H–M)** HUVECs were classified as shRNA knockdown (ShplexinA1) or control (NC) group. **(H)** The proliferative ability of HUVECs were detected by CCK8 assay. **(I)** The optical microscopic image (up) and the fluorescence microscopic image (down). Representative photographs of migration **(J)** and tube formation **(L)**, and quantitative analysis of migration **(K)** and tube formation **(M)** are shown. Data are expressed as mean ± SD (n = 3, *P < 0.05, **P < 0.01).

To further detect what is the mechanism of stress on tumour angiogenesis, we assessed VEGF expression in HUVECs after incubated with ISO. HUVECs displayed markedly increased VEGF content following ISO treatment (**Figure 3A**). This suggests that stress can promote the VEGF autocrine of vascular endothelial cells. Then we used human recombinant VEGF165 to incubate HUVECs to detect changes in downstream molecules. Exposure to VEGF165 increased the VEGFR2 (**Figure 3B**) and plexinA1 (**Figure 3C**) mRNA level within HUVECs depending on its concentration. Also, VEGF165 treatment increase protein levels of VEGFR2 and plexinA1. (**Figure 3D**)

As suggested by these findings, ISO treatment significantly promoted the VEGF autocrine, and VEGF further increases the expression of VEGFR2 and plexinA1. To further confirm this process, we use ISO and ICI, the agonist and the specific blockers of β2-AR to incubate HUVECs respectively. The β2-AR expression were obtained from western-blotting analysis. (**Figure 3F**) Further, our findings revealed that VEGFR2 and plexinA1 upregulated by ISO stimulation, whereas ICI treatment reduced mRNA (**Figure 3E**) and proteins (**Figure 3F**) expression of VEGFR2 and plexinA1. Cabozantinib, a selective inhibitor of VEGFR2 was selected in this study to elucidate the role of VEGFR2 and plexinA1 in ISO promote angiogenesis. Study has shown the IC50 of cabozantinib almost >400 nM (18). Hence, we selected 40 nM cabozantinib as research concentrations in HUVEC and hCMEC/D3. Our results indicated that blocking VEGFR2, ISO act on vascular endothelial cells through plexinA1. (**Figure 3G**) Collectively, our observations showed that ISO/β2-AR-VEGF-VEGFR2/plexinA1 signaling served as a regulator in mediating the tumour angiogenesis.

To further verify the function of plexinA1, we introduced the shplexinA1 interference sequence or control shNC in HUVECs. The transfection efficiency was approximately 76% based on the percentage of GFP-positive cells. (**Figure 3I**) Then, CCK8 and transwell assay was applied in combination with tube formation assay for evaluating the impact of plexinA1 on HUVECs by interfering plexinA1 expression. We found shplexinA1 can significantly decrease the ability of proliferation (**Figure 3H**), migration (**Figures 3J, K**) and tube formation (**Figures 3L, M**) in HUVECs. Collectively, our findings indicated plexinA1 is indispensable in tumour angiogenesis.

### Isoprenaline Promotes HUVECs Activation by plexinA1/VEGFR2-JAK2-STAT3 Signaling Pathway

**Figure 4:**
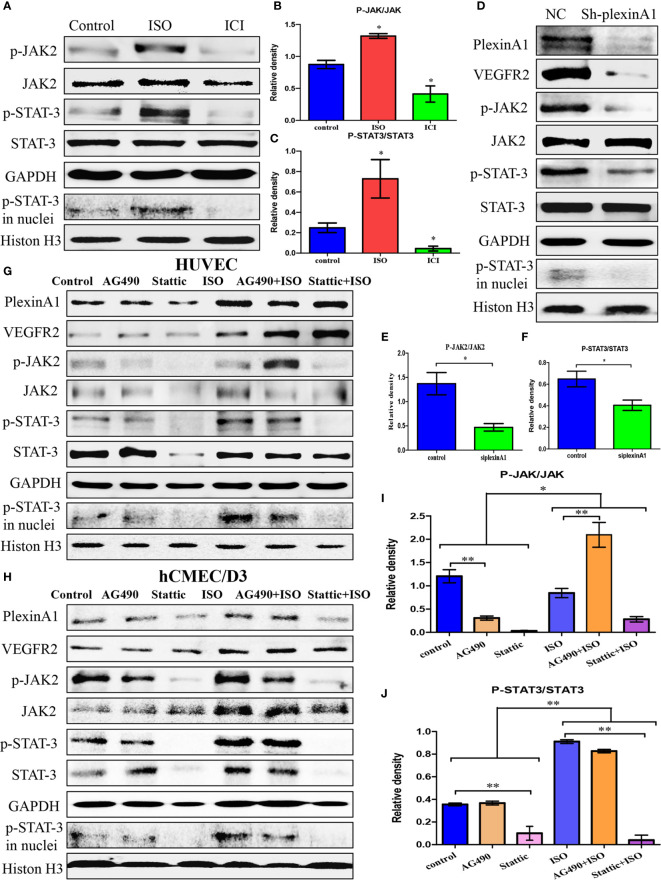
Isoprenaline promotes plexinA1/VEGFR2 expression *via* JAK2-STAT3 pathway. **(C)**HUVECs were treated with ISO or ICI. **(A)** The protein levels of JAK2/STAT3 were analyzed. The effect of isoprenaline on p-JAK2/JAK2 **(B)** and p-STAT3/STAT3 **(C)** expression. **(D–F)** HUVECs were classified as NC and ShplexinA1 group. **(D)** PlexinA1, VEGFR2 and JAK2/STAT3 at protein levels were evaluated by western blots. The effect of ShplexinA1 on p-JAK2/JAK2 **(E)** and p-STAT3/STAT3 **(F)** expression. **(G–J)**: HUVECs and hCMEC/D3 were treated with AG-490 or stattic, and then incubated in the medium containing 0, 10uM isoprenaline for additional 12h. **(G)** PlexinA1, VEGFR2 and JAK2/STAT3 at protein levels in HUVECs were evaluated by western blots. **(H)** PlexinA1, VEGFR2 and JAK2/STAT3 at protein levels in hCMEC/D3 were evaluated by western blots. The effect of JAK2-STAT3 pathway inhibitors and isoprenaline on p-JAK2/JAK2 **(I)** and p-STAT3/STAT3 **(J)** expression. Data are expressed as mean ± SD (n = 3, *P < 0.05, **P < 0.01).

We found that ISO not only promoted the expression of plexinA1 and VEGFR2, but also activated the JAK2-STAT3 pathway. ISO mainly promoted the phosphorylation of JAK2 and STAT3, and increased the translocation of p-STAT3. (**Figures 4A–C**) To further detect the mechanism of between plexinA1 and upstream and downstream signals, we tested the effects of shplexinA1 on JAK2-STAT3 pathway by western blots. Inhibition of plexinA1 strongly decreased the expression of VEGFR2, and phosphorylation of JAK2 and STAT3. At the same time, inhibition of plexinA1 also decreased the translocation of p-STAT3. (**Figures 4D–F**) These results suggested that ISO-plexinA1/VEGFR2-JAK2-STAT3 signaling may be related with tumour angiogenesis. In order to verify the hypothesis, AG-490, a phosphorylation inhibitors of JAK2, as well as stattic, a phosphorylation inhibitors of STAT3 were used respectively to detect that isoprenaline promote activation of HUVECs. PlexinA1 and VEGFR2 protein expression was not affected by JAK2-STAT3 pathway inhibitors, but could be activated by isoprenaline. (**Figure 4G**) Isoprenaline had no effect on the expressions of JAK2 and STAT3, but increased the expressions of p-JAK2, p-JAK3 and the translocation of p-STAT3 in HUVECs. (**Figures 4G, I, J**) Similar results were observed in hCMEC/D3 cells at protein levels. (**Figure 4H**) These results indicated that isoprenaline could promote plexinA1 and VEGFR2 protein expression, and then activate JAK2-STAT3 pathway.

All together, these results suggested that chronic stress promote plexinA1/VEGFR2-JAK2-STAT3 in VECs to promote angiogenesis.

## Discussion

Chronic stress is related to the abnormally continuous activation of hypothalamus-pituitary-adrenal axis and the excitation of sympathetic nervous system, resulting in increased release of catecholamine, especially norepinephrine and epinephrine. Catecholamine can regulate multiple biological behaviors of tumour cells by influence proliferation, adhesion, migration, and invasion (19). Also, high catecholamine level has been detected within tumour microenvironment (20). The tumour microenvironment is crisscrossed with blood and lymph vessels. Blood vessel exerts a vital part during tumour development. It is urgent to understand the catecholamines function in tumour angiogenesis.

The α- and β-adrenergic receptor families function to mediate the catecholamine biological functions. Of the catecholamine receptors, β2-AR is the most typical one. Catecholamine can activate β2-AR, which indicates dismal prognosis. Our previous study found that β2-AR agonist affected EMT of gastric cancer cells through stat3-CD44 (21). Other researchers have also found that isoprenaline affects gastric cancer cell EMT through β2-AR-HIF-1α-Snail signal transduction pathway, which thus affects gastric cancer migration and invasion (22). Besides, tumour growth and angiogenesis were inhibited by β2-AR gene knockout in prostate cancer (23). By adopting the animal models of ovarian cancer, Thaker and colleagues identified the vital part of β2-AR in stress-mediated tumour progression through enhancing angiogenesis (4). Another research reported that chronic stress influences tumour angiogenesis through the secretion of VEGF through activating β2-AR-HIF-1α pathway. With prominent expression of pro-angiogenic growth factors like VEGF, the tumour microenvironment entered a “highly vascularized” state, which promotes the malignant progression of the tumour. In this study, we use selective β2-AR agonists and blockers to discover that chronic stress regulates the tumour angiogenesis pathophysiology.

**Figure 5:**
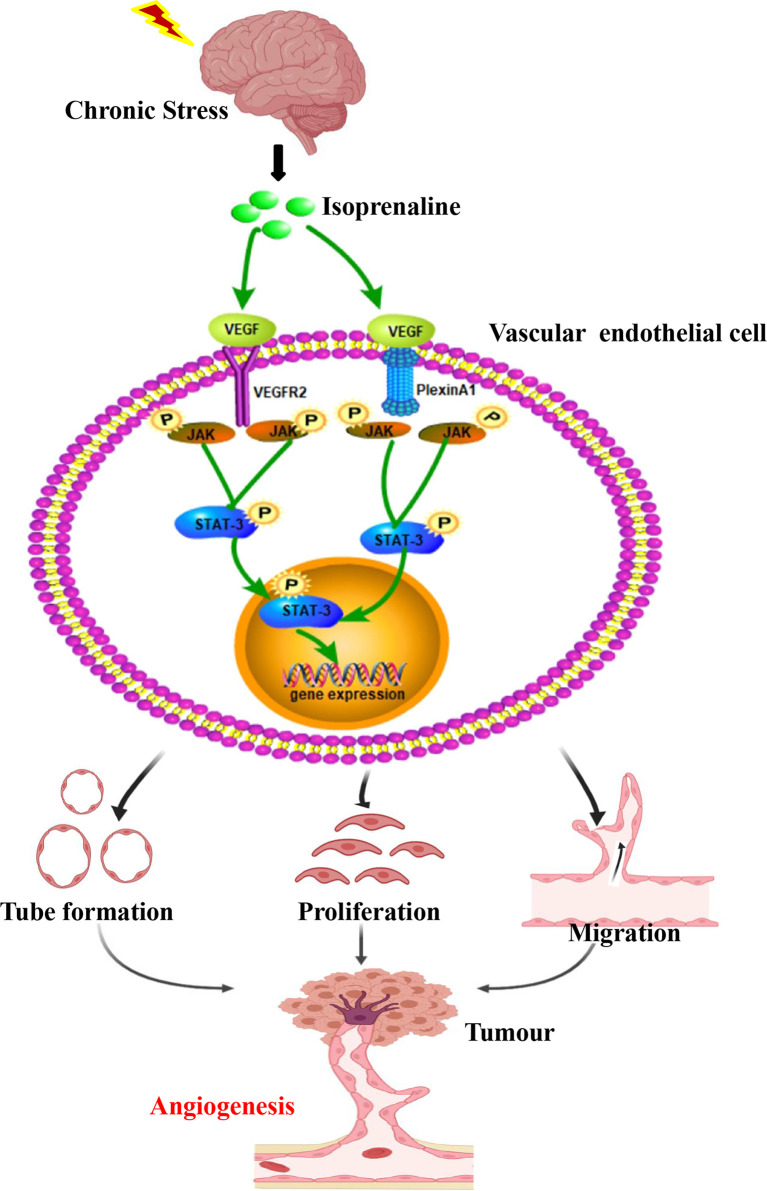
Chronic stress induced tumour angiogenesis. This study suggested that stress-related hormones were directly connected with tumour angiogenesis. Based on this findings, a majority of cancer cases suffered from depression and anxiety to certain degrees, which trigger body release catecholamines. The β2-AR agonist isoprenaline activated VEGF autocrine of vascular endothelial cells, which subsequently recognized and activated their receptors VEGFR2. PlexinA1 is necessary in the process of VEGF activated VEGFR2 and further affected the ability of proliferation, migration and tube formation of VECs through activating JAK2-STAT3 signaling pathway. Thus, chronic stress promote plexinA1/VEGFR2-JAK2-STAT3 in VECs to promote tumor angiogenesis.

Based on this study, chronic stress regulates the tumour angiogenesis pathophysiology (**Figure 5**). Besides, chronic stress causes enhanced of intratumoral perfusion values. Further, we find chronic stress increases tumour angiogenesis by increasing HUVECs proliferation, migration, along with tube formation. Based on the above findings, chronic stress exerts a vital part in tumour angiogenesis. VEGF mainly functions to regulate angiogenesis, which is also a candidate autocrine growth factor of VECs within a variety of cancers (24). Indeed, we also found that chronic stress increases VEGF autocrine in VEC, which could potentially increase tumour angiogenesis.

In this study, exposure to human recombinant VEGF165 promoted plexinA1 and VEGFR2 levels within HUVECs. Our finding shows that VEGFR2 and plexinA1 exert an important part in isoprenaline increase angiogenesis. It is well-known VEGFR2’s role in angiogenesis. Ferrara and colleagues reported that, VEGFR2 mediated the VEGF function, and the former existed in tumour-related VECs (25). PlexinA1 and VEGFR2 can constitute a receptor complex, thus exerting diverse impacts on diverse cardiac tube sites (4). It is significant to illustrate the role of plexinA1 during angiogenesis. Then, our results suggested that, plexinA1 knockdown within HUVECs decreased the proliferation, migration and tube formation. It gives us valid support that plexinA1 is indispensable in the VEGF-VEGFR2 signaling pathway in chronic stress-induced tumour angiogenesis.

JAK2-STAT3 pathway is one of the most important cell signal transduction pathways. Some studies have shown that JAK-STAT3 pathway plays a key role in endothelial cell activation and can increase the cell proliferation and adhesion in endothelial cells (26, 27). For example, activation of JAK-STAT3 pathway is essential for IL-6-mediated proinflammatory response of HUVECs (28). We found that JAK2-STAT3 signaling pathway participate in the activation of endothelial cells in stress-induced tumour progression, which happens immediately after the activation of plexinA1 and VEGFR2 by isoprenaline.

There are two limitations to this study. First, we did not use the hormone of tumor microenvironment to confirm its effect on blood vessels. Second, We did not identify the protein-protein interaction between VEGFR2 and plexinA1.

As suggested in this work, the stress-associated hormones are directly linked with angiogenesis in gastric cancer. They promote the autocrine of VEGF and increase the levels of VEGFR2 and plexinA1, later activate JAK2-STAT3 pathway in VECs. As a result, more strategies targeting plexinA1 and novel treatment against angiogenesis should be developed in future works to treat gastric cancer.

## Data Availability Statement 

The original contributions presented in the study are included in the article/**Supplementary Material**. Further inquiries can be directed to the corresponding authors.

## Ethics Statement 

The Ethics Committees of Chengde Medical University approved the patients’ consents for participation and blood sample use in the present work (2020003). The patients/participants provided their written informed consent to participate in this study. All animal studies were conducted with approval of the Institutional Animal Care and Use Committee of General Hospital of People’s Liberation Army (2015-X11-09).

## Author Contributions

YJL and HZZ performed the research and wrote the manuscript; YHL and XZ as the corresponding author reviewed the manuscript, made significant revisions and contributed to the reagents; YZZ, QX, LL, and XL contributed to discussions about the manuscript; HBZ and SZ participated in collecting tissues samples; YZ and YL carried out experiments and analyzed data. All authors contributed to the article and approved the submitted version.

## Funding

The present study was funded by the National Natural Science Foundation of China (81672462, 81703001), the Natural Science Foundation of Hebei Province, China (H2019406073, H2020406008), the University level research project of Chengde Medical College (202007, KY2021037), the Health Commission Foundation of Hebei Province (20200355), Technology Innovation Guidance Project-Science and Technology Work Conference of Hebei Provincial Department of Science and Technology, the Key Subjects (Pathology and Pathophysiology) at Colleges and Universities of Hebei Province.

## Conflict of Interest

The authors declare that the research was conducted in the absence of any commercial or financial relationships that could be construed as a potential conflict of interest.

## Publisher’s Note

All claims expressed in this article are solely those of the authors and do not necessarily represent those of their affiliated organizations, or those of the publisher, the editors and the reviewers. Any product that may be evaluated in this article, or claim that may be made by its manufacturer, is not guaranteed or endorsed by the publisher.
