# Supplementary material for: Chronic Stress Activates PlexinA1/VEGFR2-JAK2-STAT3 in Vascular Endothelial Cells to Promote Angiogenesis
Source: Front Oncol. 2021 Aug 16;11:709057. doi: 10.3389/fonc.2021.709057 (PMC8415364; doi:10.3389/fonc.2021.709057)
Supplement: Supplementary file 1 [file Table_1.docx]

# Table S1. The results of Self-Rating Anxiety and Self-Rating Depression in 215 patients with different types of cancer

| **Cancer types** | **SAS** | **SDS** | **Cancer types** | **SAS** | **SDS** | **Cancer types** | **SAS** | **SDS** | **Cancer types** | **SAS** | **SDS** |
| --- | --- | --- | --- | --- | --- | --- | --- | --- | --- | --- | --- |
| Hypopharyngeal cancer | 50 | 73 | Endometrial cancer | 41 | 53 | Glottic carcinoma | 44 | 64 | Lung cancer | 40 | 66 |
| Ovarian malignancy | 39 | 58 | Prostate cancer | 44 | 63 | Cervical cancer | 39 | 41 | Left breast cancer | 51 | 65 |
| Lung cancer | 49 | 63 | Tonsillar malignancy | 53 | 71 | Lung cancer | 41 | 59 | Endometrial cancer | 37 | 42 |
| Bilateral ovarian cancer | 38 | 48 | Cervical cancer | 46 | 61 | Ovarian cancer | 53 | 70 | Rectal cancer | 40 | 58 |
| Endometrial cancer | 34 | 51 | Squamous cell carcinoma | 49 | 55 | Oropharyngeal cancer | 38 | 46 | Rectal cancer | 53 | 64 |
| Small cell lung cancer | 31 | 55 | Glioblastoma | 44 | 63 | Laryngeal cancer | 45 | 45 | Liver malignancy | 44 | 53 |
| Tonsillar malignancy | 46 | 68 | Left lung adenocarcinoma | 41 | 64 | Nasopharyngeal cancer | 41 | 55 | Cholangiocarcinoma | 39 | 51 |
| Small cell lung cancer | 63 | 75 | Renal cancer | 50 | 56 | Right neck adenocarcinoma | 46 | 61 | Pancreatic head cancer | 45 | 61 |
| Multiple myeloma | 31 | 48 | Endometrial cancer | 33 | 53 | Endometrial cancer | 49 | 55 | Liver malignancy | 44 | 43 |
| Colon cancer | 44 | 50 | Rectal cancer | 35 | 46 | Left lung cancer | 47 | 67 | Liver cancer | 38 | 43 |
| Cholangiocarcinoma | 45 | 73 | Colon cancer | 33 | 44 | Gastric cancer | 59 | 66 | Post operation ovarian cancer | 45 | 75 |
| Gallbladder carcinoma | 46 | 59 | Colon cancer | 44 | 66 | Breast cancer | 33 | 49 | Colon cancer | 43 | 55 |
| Rectal cancer | 66 | 69 | Sigmoid colon | 46 | 73 | Colon cancer | 38 | 53 | Rectal cancer | 33 | 42 |
| Bone cancer | 41 | 55 | Ileocecal cancer | 58 | 61 | Gastric cancer | 50 | 54 | Primary liver cancer | 57 | 85 |
| Esophagus cancer | 75 | 75 | Pancreatic cancer | 49 | 66 | Rectal cancer | 38 | 50 | Post operation pancreas cancer | 42 | 68 |
| Liver cancer | 34 | 49 | Rectal cancer | 39 | 51 | Rectal cancer | 35 | 56 | Liver space-occupying lesion | 50 | 58 |
| Lung cancer | 34 | 46 | Lung cancer | 38 | 44 | Rectal cancer | 41 | 48 | Rectal cancer | 53 | 65 |
| Prostate cancer | 36 | 49 | Non-hodgkin lymphoma | 36 | 46 | Colon cancer | 43 | 54 | Post operation gastric cancer | 45 | 78 |
| Small cell cancer of right lung | 33 | 48 | Small cell cancer of left lung | 35 | 44 | B-cell lymphoma | 46 | 41 | Post operation left lung cancer | 46 | 60 |
| Colon cancer | 46 | 45 | Left lung adenocarcinoma | 43 | 40 | Small cell cancer of left lung | 46 | 56 | Liver cancer | 45 | 66 |
| Squamous cell carcinoma | 53 | 64 | Left lung adenocarcinoma | 53 | 49 | Colon cancer | 46 | 60 | Lung cancer | 48 | 56 |
| Right lung adenocarcinoma | 53 | 64 | Small cell cancer of right lung | 35 | 53 | Lymphoma | 54 | 56 | Left lung cancer | 49 | 55 |
| Small cell cancer of right lung | 59 | 54 | Left lung adenocarcinoma | 41 | 48 | Right lung adenocarcinoma | 38 | 51 | Right lung cancer | 51 | 85 |
| Terminal lung cancer | 44 | 60 | Small cell cancer of left lung | 33 | 46 | Right lung adenocarcinoma | 38 | 45 | Right lung adenocarcinoma | 41 | 55 |
| Small cell cancer of right lung | 43 | 41 | Left lung adenocarcinoma | 43 | 49 | Left lung cancer | 45 | 48 | Left lung adenocarcinoma | 43 | 47 |
| Right lung adenocarcinoma | 36 | 49 | Right lung adenocarcinoma | 41 | 49 | Left lung adenocarcinoma | 43 | 55 | Breast cancer | 63 | 66 |
| Left lung cancer of low grade | 79 | 75 | Breast cancer | 43 | 44 | Left lung cancer | 64 | 80 | Lung cancer | 31 | 40 |
| Left lung adenocarcinoma | 44 | 44 | Left lung cancer | 50 | 44 | Left lung adenocarcinoma | 50 | 66 | Lung cancer | 33 | 51 |
| Right lung adenocarcinoma | 36 | 48 | Breast cancer | 35 | 45 | Lung cancer | 41 | 45 | Thyroid nodule | 34 | 36 |
| Esophagus cancer | 41 | 50 | Left lung adenocarcinoma | 68 | 64 | Right lung adenocarcinoma | 41 | 45 | Lung cancer | 37 | 50 |
| Lung cancer | 38 | 44 | Lung cancer | 53 | 71 | Lung cancer | 59 | 69 | Lung cancer | 38 | 54 |
| Right lung cancer | 46 | 54 | Right lung cancer | 45 | 59 | Lung cancer | 43 | 51 | Breast cancer | 35 | 51 |
| Breast cancer | 45 | 58 | Breast cancer | 41 | 60 | Breast cancer | 33 | 46 | Breast cancer | 48 | 51 |
| Lung cancer | 38 | 50 | Left lung adenocarcinoma | 53 | 68 | Breast cancer | 40 | 59 | Esophagus cancer | 39 | 43 |
| Lung cancer | 38 | 54 | Breast cancer | 36 | 49 | Breast cancer | 45 | 56 | Breast cancer | 69 | 42 |
| Breast cancer | 33 | 31 | Esophagus cancer | 39 | 44 | Breast cancer | 45 | 55 | Breast cancer | 51 | 50 |
| Right lung adenocarcinoma | 44 | 48 | Esophagus cancer | 39 | 48 | Lung cancer | 45 | 58 | Lung cancer | 61 | 48 |
| Breast cancer | 41 | 38 | Esophagus cancer | 39 | 41 | Breast cancer | 54 | 75 | Breast cancer | 62 | 53 |
| Lung cancer | 36 | 49 | Breast cancer | 34 | 44 | Breast cancer | 46 | 71 | Breast cancer | 52 | 42 |
| Bone cancer | 43 | 76 | Lung cancer | 34 | 48 | Breast cancer | 45 | 59 | Esophagus cancer | 54 | 43 |
| Breast cancer | 40 | 55 | Lung cancer | 39 | 50 | Breast cancer | 43 | 73 | Breast cancer | 67 | 52 |
| Liver cancer | 53 | 66 | Breast cancer | 39 | 49 | Breast cancer | 41 | 48 | Lung cancer | 38 | 42 |
| Rectal cancer | 41 | 54 | Ovarian cancer | 36 | 36 | Breast cancer | 48 | 55 | Rectal cancer | 66 | 58 |
| Lung cancer | 51 | 45 | Pancreatic cancer | 45 | 61 | Breast cancer | 49 | 60 | Osteosarcoma | 55 | 46 |
| Gastric cancer | 56 | 61 | Stomach cancer | 58 | 71 | Prostate malignancy | 44 | 61 | Left lung cancer | 44 | 58 |
| Glioblastoma | 50 | 75 | Sigmoid colon cancer | 59 | 65 | Lung cancer | 36 | 58 | Rectal cancer | 38 | 50 |
| Left breast cancer | 40 | 53 | Lung cancer | 38 | 54 | Esophagus cancer | 55 | 66 | Left lung squamous cell carcinoma | 33 | 41 |
| Right lung squamous cell carcinoma | 41 | 54 | Left lung squamous cell carcinoma | 33 | 44 | Left lung squamous cell carcinoma | 48 | 65 | Left lung squamous cell carcinoma | 39 | 49 |
| Hypopharyngeal squamous cell carcinoma | 52 | 62 | Cervical squamous cell carcinoma | 45 | 65 | Cervical squamous cell carcinoma | 40 | 65 | Left lung squamous cell carcinoma | 63 | 80 |
| Cervical squamous cell carcinoma | 52 | 65 | Cervical  squamous cell carcinoma | 41 | 68 | Post operation right colon cancer | 60 | 67 | Left lung squamous cell carcinoma | 39 | 44 |
| Post operation left lung adenocarcinoma | 36 | 55 | Spindle cell carcinoma of left knee | 45 | 45 | Right lung squamous cell carcinoma | 50 | 76 | Left lung squamous cell carcinoma | 38 | 51 |
| Left lung squamous cell carcinoma | 39 | 43 | Left lung squamous cell carcinoma | 38 | 51 | Left lung squamous cell carcinoma | 50 | 55 | Right lung squamous cell carcinoma | 43 | 50 |
| Left lung squamous cell carcinoma | 45 | 55 | Left lung squamous cell carcinoma | 39 | 39 | Gingival squamous cell carcinoma | 30 | 45 | [Head](C:/%E5%AE%89%E8%A3%85%E8%BD%AF%E4%BB%B6/qq/Youdao/Dict/6.3.69.8341/resultui/frame/javascript:void(0);) [and](C:/%E5%AE%89%E8%A3%85%E8%BD%AF%E4%BB%B6/qq/Youdao/Dict/6.3.69.8341/resultui/frame/javascript:void(0);) [neck](C:/%E5%AE%89%E8%A3%85%E8%BD%AF%E4%BB%B6/qq/Youdao/Dict/6.3.69.8341/resultui/frame/javascript:void(0);) [squamous](C:/%E5%AE%89%E8%A3%85%E8%BD%AF%E4%BB%B6/qq/Youdao/Dict/6.3.69.8341/resultui/frame/javascript:void(0);) [cell](C:/%E5%AE%89%E8%A3%85%E8%BD%AF%E4%BB%B6/qq/Youdao/Dict/6.3.69.8341/resultui/frame/javascript:void(0);)  [carcinoma](C:/%E5%AE%89%E8%A3%85%E8%BD%AF%E4%BB%B6/qq/Youdao/Dict/6.3.69.8341/resultui/frame/javascript:void(0);) | 40 | 56 |
| Left lung squamous cell carcinoma | 58 | 68 | Cervical squamous cell carcinoma | 44 | 56 | Cervical squamous cell carcinoma | 40 | 49 |  |  |  |
